# Patterns and Outcomes of Obesity Using Body Mass Index in Patients Hospitalized with Acute Cardiovascular Disorders: A Retrospective Analysis of 7284 Patients in a Middle Eastern Country

**DOI:** 10.3390/jcm12237263

**Published:** 2023-11-23

**Authors:** Abdul Rehman Abid, Ayman El-Menyar, Rajvir Singh, Mohamed Gomaa, Said Habib, Ahmed Shaaban Abdelrahman, Nidal Asaad, Awad AlQahtani, Hassan Al-Thani, Hajar AlBinali, Jassim Al Suwaidi

**Affiliations:** 1 Cardiology Department, Heart Hospital, Hamad Medical Corporation (HMC), Doha P.O. Box 3050, Qatar; aabid1@hamad.qa (A.R.A.); mgad@hamad.qa (M.G.); shabib1@hamad.qa (S.H.); nasaad@hamad.qa (N.A.); aalqahtani7@hamad.qa (A.A.); hajar@hamad.qa (H.A.); jalsuwaidi@hamad.qa (J.A.S.); 2 Vascular Surgery, Clinical Research, Hamad Medical Corporation (HMC), Doha P.O. Box 3050, Qatar; 3 Department of Clinical Medicine, Weill Cornell Medical School, Doha P.O. Box 24144, Qatar; 4 Cardiovascular Research, Heart Hospital, Hamad Medical Corporation (HMC), Doha P.O. Box 3050, Qatar; rsingh@hamad.qa; 5 Cardiology Department, Al Wakrah Hospital, Hamad Medical Corporation (HMC), Al Wakrah P.O. Box 82228, Qatar; aabdelrahman3@hamad.qa; 6 Department of Surgery, Vascular Surgery, HMC, Doha P.O Box 3050, Qatar; althanih@hotmail.com

**Keywords:** obesity, diabetes mellitus, acute coronary syndrome, myocardial infarction, BMI, metabolic syndrome, cardiovascular, congestive heart failure, AF

## Abstract

Background: We aimed to study the presentation and in-hospital outcomes of obese patients hospitalized for cardiovascular diseases (CVDs) in a Middle Eastern country. Methods: This retrospective study included patients admitted to the Heart Hospital between 2015 and 2020. Patients were divided according to their body mass index (BMI): Group I (BMI 18.5–24.9), Group II (BMI 25–29.9), and Group III (BMI ≥ 30), by applying one-way ANOVAs and chi-square tests. The obese group (BMI ≥ 30) was graded and compared (Grade I (BMI 30–34.9), Grade II (BMI 35–39.9), and Grade III (BMI ≥ 40)). Results: There were 7284 patients admitted with CVDs (Group I (29%), Group II (37%), and Group III (34%)). The mean age was higher in Group III than Groups I and II (*p* < 0.001). Male sex was predominant in all groups except for morbid obesity (Grade III), in which females predominated. Diabetes mellitus (DM), hypertension, and dyslipidemia were more common in Group III. Chest pain was more common in Group II, while shortness of breath was more evident in Group III (*p* < 0.001). Group II had more ST-elevation myocardial infarction (STEMI), followed by Group I (*p* < 0.001). Atrial fibrillation (AF) was observed more frequently in Group III (*p* < 0.001). Congestive heart failure (CHF) was common in Group III (19%) (*p* < 0.001). In the subanalysis, (Grade I (62%), Grade II (22.5%), and Grade III (15.5%)), Grade I had more STEMI, whereas AF and CHF were higher in Grade III (*p* < 0.001). Percutaneous Coronary Intervention was performed less frequently in Grade III (*p* < 0.001). In-hospital mortality was higher in Grade III (17.1%), followed by Grades II (11.2%) and I (9.3%) (*p* < 0.001). Conclusions: In this study, one third of the hospitalized CVS patients were obese. AF and CHF with preserved EF were the most common cardiovascular presentations in obese patients. In patients with CVDs, obesity was associated with higher rates of comorbidities and in-hospital mortality. However, obesity measured by BMI alone was not an independent predictor of mortality in obese cardiac patients.

## 1. Introduction

Cardiovascular disease (CVD) is acknowledged as the primary global cause of mortality, responsible for 32% of all deaths or a total of 20.5 million deaths in 2021 [1]. Coronary artery disease (CAD) is influenced by obesity, an independent risk factor [2]. The prevalence of obesity continues to escalate, as evidenced by data from the National Health and Nutrition Examination Surveys (NHANES), indicating a rise in obesity prevalence among adults in the USA from 30.5% to 42.4%, with severe obesity nearly doubling from 4.7% to 9.2% [3]. In the Western world, the prevalence of obesity is 36.5% [4], while the Middle East reports prevalence rates of overweight and obese individuals ranging from 23.5% to 62.1% and 14.5% to 40.6%, respectively [5]. 

Recent statistics reveal that 43% and 24% of coronary revascularization procedures were performed in overweight and obese patients, respectively [6]. The mechanisms implicated in atherosclerosis development in obesity include abnormalities in lipid metabolism, insulin resistance, inflammation, endothelial dysfunction, and adipokine imbalance [7]. Obesity is also linked to type 2 diabetes, hypertension, heart disease, sleep apnea, and certain types of cancer [8]. Type 2 diabetes affects 18.5% of adults with obesity compared to 5.4% with normal weight, peaking at 23.2% in those with severe obesity [9]. 

A positive association between high body mass index (BMI) and coronary artery disease (CAD) risk is supported by findings that every 1 kg/m^2^ increase in body mass index leads to a 5–7% increase in CAD incidence across all BMI categories [10]. Obese patients exhibit a significantly higher risk of CAD even after accounting for age, sex, physical activity, and smoking [11]. 

Excess adiposity was found to be responsible for 23% of CHD in men and 15% in women, according to the Framingham study [12]. The duration of obesity correlates with an increased risk of cardiovascular disease mortality, with a clear dose-dependent pattern; for every two additional years with obesity, CVD mortality risk rises by 7% [13]. Compared to individuals with a normal BMI, obesity is associated with a significantly heightened risk of CVD morbidity and mortality [14]. Overweight and obese individuals have a greater incidence of CVD events compared to those with normal weight [15]. 

Interestingly, previous studies showed that obese patients display better survival rates than normal-weight and underweight individuals, a phenomenon known as the “obesity paradox” [16,17]. Overweight and obese patients with CHD also exhibit a lower risk of total and cardiovascular mortality compared to underweight and normal-weight CHD patients [18]. However, recent studies did not show this survival effect in patients with low ejection fraction heart failure or acute coronary syndrome when using alternative anthropometric measurements rather than weight or adjustment for other predictors [19,20].

A meta-analysis of patients with acute coronary syndrome demonstrated that overweight, obese, and severely obese patients have significantly lower mortality rates compared to those with a normal BMI [21]. Similarly, overweight and obese patients with congestive heart failure (CHF) experience reduced CVD mortality (19% and 40%, respectively) and all-cause mortality (16% and 33%, respectively) compared to CHF patients with normal BMI [22]. The low-BMI group, consisting of underweight or normal-weight individuals, demonstrated the highest rates of adverse events, mortality, and rehospitalizations, while the overweight group exhibited the lowest rates [23]. 

Given the high prevalence of obesity in the Middle East, there is a need to investigate the epidemiology and impact of obesity among hospitalized CVD patients. This study aimed to assess, in our region, the effect of obesity on the presentation, diagnosis, and in-hospital outcomes of CVDs. We hypothesized that obesity has particular CVS presentations and outcomes (length of hospital stay, cardiac complications, and mortality). 

## 2. Materials and Methods

Study design and setting: This was a retrospective observational study of patients admitted to the Heart Hospital, Hamad Medical Corporation (HMC) between the 1 January 2015 and the 31 December 2019. Data were collected from the Coronary Care Unit (CCU) registry at the Heart Hospital from January 2015 to December 2019. The Heart Hospital at HMC is a tertiary hospital that caters for more than 95% of the cardiac inpatient and outpatient medical and surgical care needs of nationals and residents of Qatar. Data from each patient were collected by the patients’ physicians at the time of discharge on a predefined coded record form (CRF). Recorded data were checked and validated by a research coordinator in the department. Approximately 40% of Qatar’s population are Arabs, including Qatari nationals, and 60% are non-Arabs. 

Study population and data: The study included all adult male and female patients admitted to the cardiology department with BMI documented on admission. Patients with incomplete cardiac or BMI data were excluded. Obesity was defined according to the World Health Organization criteria [24]. After fulfilling the inclusion criteria, patients were divided into three groups according to BMI: Group I, normal weight (18.5–24.9 kg/m^2^); Group II, overweight (25–29.9 kg/m^2^); and Group III, obese (≥30 kg/m^2^). The obese group was further divided into three grades: Grade 1 (30–34.9 kg/m^2^), Grade 2 (35–39.9 kg/m^2^), and Grade 3 (≥40 kg/m^2^) [24]. Subgroup analysis of the obese cohort (III) was performed to study obesity grades based on presentation, diagnosis, and in-hospital outcomes. 

Research tools: The CRF included history, along with information regarding risk factors for CHD, indication for coronary angiography, and the angiographic findings of each patient. Selective coronary angiography was performed using Judkins catheters via the radial or femoral route as per the suitability assessed by the interventional cardiologist. Any artery with >70% luminal narrowing was stented with a drug-eluting stent.

The presence of diabetes mellitus was determined by the documentation in the patient’s previous or current medical record of a diagnosis of diabetes mellitus that had been treated with medication or insulin. The presence of hyperlipidemia was determined by a fasting cholesterol > 5.2 mmol/L in the patient’s medical record or any history of hyperlipidemia treatment by the patient’s physician. The presence of hypertension was determined by documentation in the medical record of hypertension, or if the patient was receiving treatment from their physician. Patients were divided into current cigarette smokers, past smokers with more than six months of abstinence from smoking, and those with no smoking history. The diagnosis of acute coronary syndrome was based on the final decision of the assigned consultants based on clinical, electrocardiographic, laboratory, and echocardiographic data.

Statistical analysis: Continuous variables were expressed as mean ± Standard Deviation (SD) or Interquartile Range (IQR), as appropriate, while categorical variables were expressed as frequencies and percentages. Risk factors, final diagnosis, laboratory parameters, Percutaneous Coronary Intervention (PCI) at the time of admission or prior to discharge, and complications during the hospital stay, including in-hospital mortality, were compared among Groups I–III by applying one-way analysis of variance (ANOVA) or Kruskal–Wallis (non-parametric) tests for continuous variables, and chi-square tests for categorical variables. Obese patients were reassessed by subanalysis according to their BMI (Grades I–III). Multivariate regression analysis was performed for in-hospital mortality in the overall cohort and then in each major cardiovascular presentation (AMI, CHF and AF), and also for obese patients (BMI ≥ 30); data were expressed as crude and adjusted odds ratios (ORs) and a 95% confidence interval. Data were adjusted for age, sex, diabetes mellitus and hypertension. *p* values were calculated and a two-tailed *p* value of less than or equal to 0.05 was considered statistically significant. Statistical analysis was performed using SPSS (version 22.0; SPSS, Inc.; Chicago, IL, USA) for Windows.

## 3. Results

**Figure 1:**
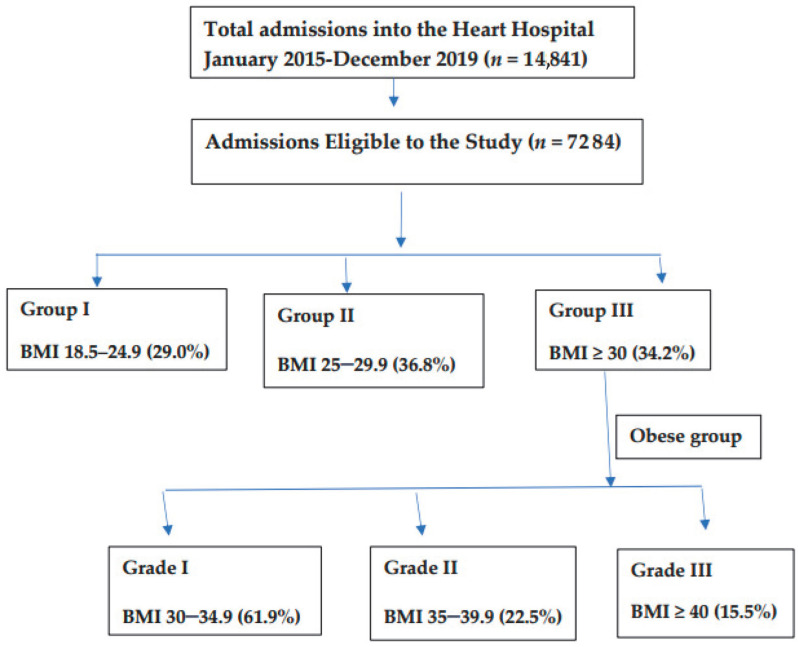
Flowchart of the study design and body mass index (BMI) groups.

There were 7284 patients admitted with CVD to the Heart Hospital who were eligible for enrollment in the study analysis (Figure 1). 

Table 1 shows the baseline characteristics of the study population. The mean age was significantly higher in Group III than in Groups I and II.

**Figure 2:**
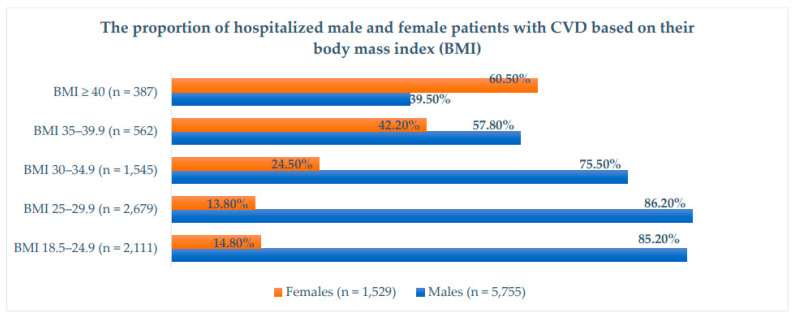
Body mass index (BMI) in males and females.

Figure 2 shows the proportion of hospitalized male and female patients with CVD based on body mass index (BMI). Male sex was dominant in all BMI groups, except in the group with morbid obesity (≥40), where female sex predominated. 

Diabetes mellitus (DM) was more prevalent (61.6%) in Group III (*p* < 0.001). Similarly, hypertension was observed in 66.4% of patients in Group III, followed by 53.5% in Group II and 44.6% in Group I (*p* < 0.001). Dyslipidemia was noted in 56.9% of Group III, 40.9% of Group II, and 36.1% of Group I (*p* < 0.001). Chronic renal failure and peripheral arterial disease exhibited a similar distribution pattern to hypertension and dyslipidemia (*p* < 0.001). Smoking was observed more frequently in Group II than in Groups I and III (*p* < 0.001).

Chest pain was observed in 69.5% of patients in Group II, 66% in Group I, and 56.5% in Group III (*p* < 0.001). Shortness of breath and palpitations were most common in Group III, followed by Groups I and II (*p* < 0.001).

**Figure 3:**
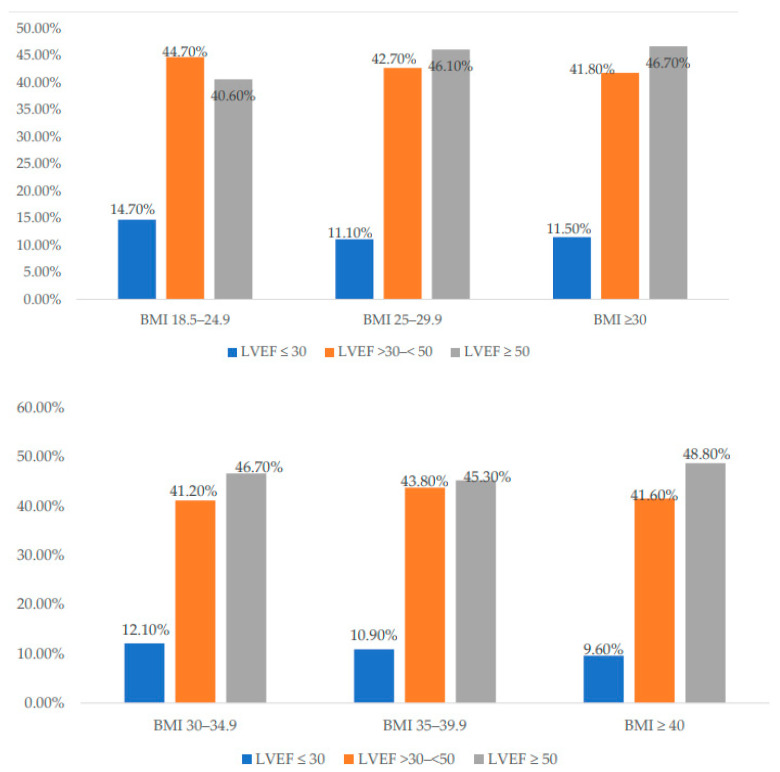
Left ventricular ejection fraction (LVEF) based on body mass index (BMI) in the cohort (upper panel) and the obese groups (lower panel).

Table 2 and Figure 3 show the laboratory parameters of the study population. Troponin levels at the time of admission were higher in Group I, followed by Group II and Group III, *p* < 003. Peak troponins were also higher in Group II, followed by Group I and Group III, *p* < 0.001. BNP level was higher in Group I, followed by Group III and Group II, *p* < 0.001. Left ventricular ejection fraction (LVEF) of <30% was observed in more patients in Group I (14.7%), followed by Group III (11.5%) and Group II (11.1%). In contrast, LVEF of >50% was observed in more patients in Group III (46.7%), followed by Group II (46.1%) and Group I (40.6%) *p* < 0.001.

Table 3 shows the final diagnosis of the study populations. The majority of patients presented with ST-segment elevation myocardial infarction (STEMI). Among Group II patients, 58.3% had STEMI, followed by Group I (57.4%) and Group III (41.6%), *p* < 0.001. A similar trend was observed in non-ST-segment elevation myocardial infarction (NSTEMI) and unstable angina, with more patients in Group II having these final diagnoses with significant *p* values. Atrial fibrillation was observed in more patients in Group III (7.3%), followed by 3.8% in Group I and 3.5% in Group II, *p* < 0.001. Congestive heart failure was the final diagnosis in 475 subjects of Group III (19%), followed by Group I (13.3%) and Group II (11.4%), *p* < 0.001. Cardiomyopathy was the final diagnosis in 9.8% of Group I, 9.3% of Group III, and 7.3% of Group II, *p* < 0.004. The mean hospital stay was 4 days in Group I and Group III versus 3 days in Group II, *p* < 0.06.

Table 3 also shows procedures performed during hospital stay. PCI at the time of admission was performed more in Group II (25.1%), followed by 24.1% in Group I and 15.4% in Group III, *p* < 0.001. Similarly, PCI before discharge was performed more in Group II (21.4%), followed by 18.8% in Group I and 17.7% in Group III, *p* < 0.003. Single vessel disease (VD) was observed more in Group II (20.2%), followed by 18.5% in Group I and 14.2% in Group III, *p* < 0.001. A similar pattern was observed in 2VD, with fewer patients (9.9%) in Group III compared to Groups II and I, *p* < 0.001. Three VD was observed more in Group I (17.7%), followed by Group II (17.1%) and Group III (13.1%), *p* < 0.001.

Atrial fibrillation (AF) during the hospital stay occurred more frequently in Group III (3.2%), followed by Group II (2.1%) and Group I (1.9%), *p* < 0.006. Ventricular fibrillation (VF) occurred more frequently in Group I than Groups II and III, *p* = 0.03. Ventricular tachycardia (VT) was also observed more frequently in Group I than in the other groups. Shock also occurred more frequently in Group I (1.9%), followed by Group II (1.8%) and Group III (1.0%), *p* < 0.02. More in-hospital mortality was observed in Group III (10.9%), followed by Group II (7.7%) and Group I (7.6%), *p* < 0.001 (Table 4).

Subgroup Analysis of the obese group: The obese cohort (BMI ≥ 30) was graded according to their BMI (61.9% Grade I, 22.5% Grade II, and 15.5% Grade III). Table 5 shows the baseline characteristics of the obese population. The mean age was higher in Grade III than in Grades I and II (*p* < 0.001). DM was observed in 71.1%, 66.5%, and 57.3% of patients in Grades III, II, and I, respectively (*p* < 0.001). Similarly, hypertension was observed in 80.9%, 71.9%, and 60.8% of patients in Grades III, II, and I, respectively (*p* < 0.001). Dyslipidemia was noted in 65.5%, 63.3%, and 52.1% of patients in Grades II, III, and I, respectively (*p* < 0.001). Chronic renal failure exhibited a distribution pattern similar to that of DM and hypertension (*p* < 0.001). Smoking was observed more frequently in Grade I than in Grades II and III (*p* < 0.001). Chest pain was the most common symptom on admission, followed by shortness of breath and palpitations. Chest pain was observed in 60.9%, 53.2%, and 43.7% of patients in Grades I, II, and III, respectively (*p* < 0.001). Shortness of breath was the most common symptom in Grade III, followed by Grades II and I (*p* < 0.001).

Table 6 and Figure 3 show the laboratory parameters of the study population. Troponin levels at the time of admission were higher in Grade I, followed by Grades II and III, *p* < 0.07. Peak troponins were also higher in Grade I, followed by Grades II and III, *p* < 0.007. LVEF was <30% in Grade I (12.1%), followed by Grades II (10.9%) and III (9.6%).

Table 7 shows the final diagnoses of the study population. Among Grade I patients, 47.2% had STEMI, followed by Grade II (37.2%) and Grade III (25.6%), *p* < 0.001. A similar trend was observed for NSTEMI and unstable angina, with more patients in Grade I presenting with NSTEMI or unstable angina than in the other grades. Atrial fibrillation was observed in 11.6%, 7.5%, and 6.1% of the patients in Grades III, II, and I, respectively (*p* < 0.001). Congestive heart failure was the more frequent diagnosis in Grade III (27.6%), followed by 16% and 12.5% in Grades I and II, respectively (*p* < 0.001).

Procedures performed during hospital stay are given in Table 7. PCI procedures were less performed in morbidly obese patients, Grade III, as PCI at the time of admission was performed more in Grade I (18.5%), followed by 13.5% in Grade II and 5.7% in Grade III, *p* < 0.001. Similarly, PCI before discharge was performed more in Grade I (20.5%), followed by 14.4% in Grade II and 11.4% in Grade III, *p* < 0.003. Single vessel disease was more prevalent in Grade I (16.2%), followed by 13.2% in Grade II and 7.8% in Grade III, *p* < 0.001. A similar pattern was observed with 2VD and 3VD, with fewer patients in Grade III as compared to Grades I and II, *p* < 0.001 and *p* < 0.007, respectively.

Ventricular tachycardia (VT) during the hospital stay occurred more frequently in Grade II (2.1%), followed by Grade I (1.7%), *p* = 0.02. More in-hospital mortality was observed in Grade III (17.1), followed by 11.2% in Grade II and 9.3% in Grade I (*p* < 0.001) (Table 8).

Multivariate regression analysis: The univariate analysis shows that obesity is a significant risk factor for in-hospital mortality. A multivariate analysis was performed to check whether it is also significant after adjusting for age and sex. To assess the predictive role of obesity in in-hospital mortality, the crude odds ratio (OR) of BMI ≥ 30 was 1.47 (95% CI 1.25–1.74), *p* = 0.001, in the overall cohort, 1.46 (95% CI 1.10–1.92), *p* = 0.008, among AMI patients, 0.92 (95% CI 0.69–1.23), *p* = 0.56, among CHF patients, and 0.67 (95% CI 0.38–1.17), *p* = 0.16, for AF patients. However, after adjustment for age and sex, the significant impact of BMI disappeared (OR 1.08, 95% CI 0.90–1.29), *p* = 0.39, for AMI patients. Table 9 shows the crude and adjusted OR for predictors of mortality among obese patients only. BMI ≥ 30 was a predictor for mortality; however, after adjustment for comorbidities, this effect on mortality was eliminated.

## 4. Discussion

The present study describes the impact of obesity on the presentation and outcomes of patients hospitalized for CVDs in a Middle Eastern country. In Qatar, the rate of obesity is high (33%), with a predominance of female gender [25]. Among patients who were hospitalized at the HH, obesity was a predictor of mortality in the unadjusted model. Overweight and obese patients had a 3- or 5-fold increased risk of developing thromboembolic events [26]. Moreover, obese patients with thromboembolism had characteristic risk factors and better survival [25]. However, the mechanism of this paradox in survival remains unclear. It could be related to potential lead time bias that occurs when obese patients develop cardiovascular events earlier in their lifetime or are tested earlier for CVD than patients with normal weight, leading to earlier diagnosis and treatment and confounding differences in outcomes [27]. Moreover, the authors redefined the “obesity” paradox and replaced it with the “BMI” paradox, exploring several limitations of using BMI alone to reflect subject adiposity status [28]. Furthermore, the protective effect of obesity is no longer considered.

In this analysis, one-third of the hospitalized patients were obese, and the most common reason for admission was AF, followed by CHF, particularly with a preserved ejection fraction. The average age of patients in the obese group was higher than those of patients in the normal and overweight groups. Obese individuals were more likely to have comorbidities. 

The overweight individuals had a higher proportion of STEMI among the study cohort. A 10 kg increase in body weight increases the risk of CAD by 12% and, simultaneously, the systolic blood pressure rises by 3 mmHg and the diastolic by 2.3 mmHg consequently [29]. Furthermore, NSTEMI affects more young people, and excess weight could be one of the most important risk factors ahead of smoking. The higher the BMI, the sooner NSTEMI develops [30]. The BMI paradox was also observed in STEMI [31]. Based on the available data, obesity is an independent risk factor for STEMI at a young age [32]. Our analysis showed that obesity is a significant predictor of mortality among AMI patients; however, when we considered age and sex, this effect on mortality was eliminated. 

Park et al. [33] found a BMI paradox in patients referred for PCI in whom BMI > 30 kg/m^2^ was associated with lower mortality and risk of CV events after PCI. The investigators did not explain this paradox clearly but called for further studies. Others have suggested that in obese patients, larger vessels are treated with larger stent diameter, or this could be related to the cardio-protective effect of adipokines [33,34].

A recent meta-analysis of patients with CHD concluded that obesity is associated with a lower risk of mortality in the short term but has a higher risk after 5 years (J-shaped pattern) [35].

In the current study, PCI at the index admission was higher in the overweight group than in the obese group. Single or 2VD was more frequently observed in this group, whereas 3VD was less evident in the obese group. Notably, the number of obstructed vessels decreased with increasing BMI in the obese cohort.

Some studies showed that higher BMI is strongly associated with the risk of HF with preserved EF rather than HF with reduced EF [36]. Moreover, a few studies reported that parameters of excess adiposity (using the waist in the measures) are independently associated with HF risk; however, they concluded that these parameters generally do not add substantive risk information for HF beyond BMI [27,37,38]. Shortness of breath and palpitations were common in the obese patients; however, LVEF < 30% was more frequent in the overweight group than the obese group. In contrast, LVEF > 50% was observed more in obese patients presenting with HF symptoms (HF with preserved EF). Other studies on patients with acutely decompensated HF demonstrated an obesity paradox with CHF. This paradox was largely confined to older patients and those with reduced LV function [39]. Additionally, a report from the Cleveland Clinic found that although the obesity paradox was evident, this largely disappeared after adjustment for confounders and then was confined mainly to women in the overweight BMI range [40]. Furthermore, the risk of CHF associated with obesity was noted as independent of other risk factors, including gender as well as cardiorespiratory fitness levels [41,42,43,44].

In the present study, AF was observed more frequently in obese patients than in normal-weight and overweight patients. A meta-analysis of 16 studies demonstrated that obese individuals have a nearly 50% increased risk of AF compared with non-obese individuals [45]. Importantly, a large prospective study demonstrated an approximately 4% increase in AF risk per 1-unit increase in BMI in men and women [46]. Additionally, evidence suggests that obesity may also be a risk factor for the progression of paroxysmal AF to persistent AF, which carries a higher morbidity and mortality [47].

However, as in patients with hypertension, CHD, and CHF, overweight and obese patients with AF demonstrate an obesity paradox, with a nearly 50% reduction in CV and all-cause mortality compared with AF patients with normal BMI [48,49].

In the present study, a higher in-hospital mortality was observed in the obese group, followed by the overweight and normal-weight groups. In Grade III obese patients, mortality was higher than in Grades II and I. These results are in contrast with those of previous studies, as most have suggested that overweight and obese patients have a better prognosis. A BMI of more than 25 among AMI patients was an independent predictor of lower mortality and shorter length of stay in the USA [50]. A meta-analysis reported that overweight/mildly obese patients with CHD have a lower risk of total and CVD mortality than underweight and normal-weight CHD patients [18]. The authors attributed this paradox to the lack of discriminatory power of BMI to distinguish body fat and lean mass. A positive relationship between CVD mortality and BMI has been shown in many large-scale studies [51]. The production of adipocytokines, oxidative stress, and prothrombotic state in obese people may contribute to CAD risk in a more significant way than what has been explained with other CV risk factors [52,53,54]. On the other hand, some authors consider the “lean paradox”, in which low body fat percentage and low BMI could be a predictor of worse CVD outcomes [27,55].

Our data showed that obesity (using BMI) was not an independent predictor of mortality in AF and CHF patients. On the contrary, it was a predictor of mortality among AMI patients in an unadjusted model (crude OR 1.46).

The risk of selection bias limits the retrospective design of this study. Many subjects were admitted to the Heart Hospital without having their BMI or waist circumference recorded on admission, which led to an underestimation of the prevalence of obesity in each study group. Moreover, being a single-center study limits the generalizability of the findings. There are many limitations to using BMI alone as a predictor of outcome because it does not correctly reflect subject adiposity and its distribution, but it also varies according to gender and ethnicity, and it does not account for fitness related to the proportion of lean mass to adiposity (i.e., it cannot distinguish adipose tissue from lean body mass) [22,28,56].

The patients’ follow-up was not recorded, which is a limiting factor in addressing long-term mortality and rehospitalization. The occurrence of coronary spasm, insulin resistance, and micro-vessel CAD in young obese subjects is also an interesting area for research [57,58].

## 5. Conclusions

In this study, one-third of the hospitalized CVS patients were obese. AF and CHF with preserved EF were the most common cardiovascular presentations in obese patients. In patients with CVDs, obesity was associated with higher rates of comorbidities and in-hospital mortality. However, obesity measured by BMI alone was not an independent predictor of mortality in cardiac obese patients.

## Figures and Tables

**Table 1:** Baseline Characteristics of the study cohort.

Characteristics	Group I (BMI 18.5–24.9) *n* = 2111 (29.0%)	Group II (BMI 25–29.9) *n* = 2679 (36.8%)	Group III (BMI ≥ 30) *n* = 2494 (34.2%)	*p* Value
Mean age in years	57.8 ± 15.1	58.2 ± 13.6	62.1 ± 13.7	0.001
Females/males; n (%)	312 (15%)/1799 (85%)	369 (14%)/2310(86%)	848 (34%)/1646 (66%)	0.001
Risk factors				
Diabetes mellitus	934 (44.2%)	1313 (44.0%)	1534 (61.6%)	0.001
Hypertension	942 (44.6%)	1433 (53.5%)	1657 (66.4%)	0.001
Dyslipidemia	763 (36.1%)	1096 (40.9%)	1418 (56.9%)	0.001
Smoking	586 (27.8%)	564 (28.5%)	517 (20.7%)	0.001
Old myocardial infarction	376 (17.8%)	549 (20.5%)	552 (22.1%)	0.001
Chronic renal failure	215 (10.2%)	290 (10.8%)	416 (16.7%)	0.001
Peripheral artery disease	10 (0.5%)	10 (0.4%)	19 (0.8%)	0.14
Symptoms at the time of admission				
Chest pain	139 (66.0%)	1863 (69.5%)	1409 (56.5%)	0.001
Dyspnea	533 (25.2%)	614 (22.9%)	892 (35.8%)	0.001
Palpitation	118 (5.6%)	118 (4.4%)	182 (7.3%)	0.001
Dizziness	123 (5.8%)	133 (5.0%)	104 (4.2%)	0.04

BMI: body mass index.

**Table 2:** Laboratory and echocardiographic findings on admission.

Lab Parameter	Group I	Group II	Group III	*p* Value
Hemoglobin gm/dl median (IQR)	13.3 (11.4–14.7)	13.5 (11.8–14.8)	12.6 (10.8–14.2)	0.001
FBS mmol/L (IQR)	6.6 (5.5–9.3)	6.7 (5.6–9.3)	7.4 (5.8–10.8)	0.11
Creatinine umol/L (IQR)	92 (79–116)	93 (79–115)	95 (78–131)	0.07
Serum BNP pg/mL (IQR)	1649 (282–6266)	949 (130–3909)	1175 (277–3358)	0.001
C-reactive protein mg/L (IQR)	27 (7.5–90.0)	16.8 (5–63)	17 (7–73)	0.22
Total cholesterol mmol/L (IQR)	4.3 (3.4–5.3)	4.3 (3.5–5.2)	4.1 (3.3–5.0)	0.03
Triglyceride mmol/L (IQR)	1.4 (0.97–2.0)	1.5 (1.1–2.3)	1.5 (1.1–2.2)	0.001
LDL mmol/L (IQR)	2.6 (1.8–3.4)	2.6 (1.8–3.4)	2.3 (1.6–3.0)	0.16
Troponin ng/L (IQR)	55 (16.0–260)	49 (16.6–185)	36 (14–102)	0.003
Troponin peak ng/L (IQR)	427 (49–3188)	471 (50–3114)	118 (29–1296)	0.001
CK-MB peak U/L (IQR)	13 (5–103)	11 (5–104)	5 (5–31)	0.29
LVEF median, range	45 (35–54)	47 (38–55)	47 (37–54)	0.001

LVEF: left ventricular ejection fraction; FBS: fasting blood sugar; CK-MB: Creatine kinase-MB; LDL: low-density lipoprotein; BNP: Brain natriuretic peptide. Continuous variables are given as median and IQR: interquartile range.

**Table 3:** Diagnosis of study population and procedure performed during hospital stay.

Diagnosis	Group I	Group II	Group III	*p* Value
Unstable angina	150 (7.1%)	244 (9.1%)	221 (8.4%)	0.03
NSTEMI	563 (26.7%)	746 (27.8%)	590 (23.7%)	0.002
STEMI	1211 (57.4%)	1563 (58.3%)	1037 (41.6%)	0.001
Anterior MI	306 (14.5%)	378 (14.1%)	180 (7.2%)	0.001
Lateral MI	73 (3.5%)	93 (3.5%)	51 (2.0%)	0.003
Inferior MI	127 (6.0%)	351 (13.1%)	328 (13.1%)	0.001
Posterior MI	50 (2.4%)	55 (2.1%)	34 (1.4%)	0.04
Atrial fibrillation	81 (3.8%)	95 (3.5%)	182 (7.3%)	0.001
Stent restenosis	7 (0.3%)	27 (1.0%)	16 (0.6%)	0.02
Stent thrombosis	8 (0.4%)	12 (0.4%)	5 (0.2%)	0.30
Congestive heart failure	281 (13.3%)	305 (11.4%)	475 (19.0%)	0.001
Cardiomyopathy	207 (9.8%)	195 (7.3%)	231 (9.3%)	0.004
Shock	33 (1.6%)	28 (1.0%)	26 (1.0%)	0.18
Cardiac arrest	34 (1.6%)	30 (1.1%)	29 (1.2%)	0.27
Aortic regurgitation	8 (0.4%)	3 (0.3%)	2 (0.1%)	0.03
Aortic stenosis	26 (1.2%)	40 (1.5%)	55 (2.2%)	0.03
Mitral regurgitation	29 (1.4%)	29 (1.1%)	22 (0.9%)	0.28
Mitral stenosis	15 (0.7%)	8 (0.3%)	20 (0.8%)	0.04
Coronary angiograhic results				
Single vessel disease	391 (18.5%)	542 (20.2%)	355 (14.2%)	0.001
Two vessel disease	289 (13.7%)	392 (14.6%)	247 (9.9%)	0.001
Three vessel disease	374 (17.7%)	457 (17.1%)	326 (13.1%)	0.001
PCI at admission	508 (24.1%)	672 (25.1)%	384 (15.4%)	0.001
PCI before discharge	397 (18.8%)	572 (21.4%)	442 (17.7%)	0.003

PCI: percutaneous coronary intervention; MI: myocardial infarction; NSTEMI: non-ST-segment elevation myocardial infarction; STEMI: ST-segment elevation myocardial infarction.

**Table 4:** Complications during hospital stay.

Variable	Group I	Group II	Group III	*p* Value
Atrial fibrillation	41 (1.9%)	56 (2.1%)	81 (3.2%)	0.006
Ventricular tachycardia	45 (2.1%)	43 (1.6%)	39 (1.6%)	0.27
Ventricular fibrillation	37 (1.8%)	29 (1.1%)	23 (0.9%)	0.03
Cardiac arrest	48 (2.3%)	43 (1.6%)	41 (1.6%)	0.17
Left bundle branch block	5 (0.2%)	7 (0.3%)	13 (0.5%)	0.17
Right bundle branch block	9 (0.4%)	8 (0.3%)	13 (0.5%)	0.46
Congestive heart failure	51 (2.4%)	56 (2.1%)	54 (2.2%)	0.74
Intubation	48 (2.3%)	45 (1.7%)	50 (2.0%)	0.33
Cerebrovascular accident	7 (0.3%)	3 (0.1%)	3 (0.1%)	0.14
Shock	41 (1.9%)	49 (1.8%)	25 (1.0%)	0.02
Hospital stay in days, median (interquartile range)	4 (2.5–5.0)	3 (2–5)	4 (2–5)	0.06
Mortality	161 (7.6%)	205 (7.7%)	272 (10.9%)	0.001

**Table 5:** Subgroup analysis of the obese cohort (Baseline Characteristics).

Characteristics	Grade I (BMI 30–34.9) *n* = 1545 (61.9%)	Grade II (BMI 35–39.9) *n* = 562 (22.5%)	Grade III (BMI ≥ 40) *n* = 387 (15.5%)	*p* Value
Mean age in years	61.1 ± 13.9	63.0 ± 13.3	64.4 ± 13	0.001
Females/males	378/1167	237/325	233/154	
Risk factors				
Smoking	373 (24.1%)	111 (19.8%)	33 (8.5%)	0.001
Diabetes mellitus	885 (57.3%)	374 (66.5%)	275 (71.1%)	0.001
Hypertension	940 (60.8%)	404 (71.9%)	313 (80.9%)	0.001
Dyslipidemia	805 (52.1%)	368 (65.5%)	245 (63.3%)	0.001
Chronic renal failure	223 (14.4%)	97 (17.3%)	96 (24.8%)	0.001
Old myocardial infarction	331 (21.4%)	132 (23.5%)	89 (23.0%)	0.54
Peripheral artery disease	13 (0.8%)	3 (0.5%)	3 (0.8%)	0.77
Symptoms at the time of admission				
Chest pain	941 (60.9%)	299 (53.2%)	169 (43.7%)	0.001
Dyspnea	456 (29.5%)	240 (42.7%)	196 (50.6%)	0.001

BMI: body mass index.

**Table 6:** Laboratory and echocardiographic findings on admission for obese patients.

Parameter	Grade I	Grade II	Grade III	*p* Value
Hemoglobin value gm/dl (IQR)	13 (11–15)	12 (11–14)	11.6 (10–13)	0.001
Fasting blood sugar mmol/L (IQR)	7 (6–11)	7.4 (6–11)	8 (6–11.5)	0.51
Serum Creatinine umol/L (IQR)	94 (79–124)	96 (77–136)	100 (76–161)	0.30
Serum Brain natriuretic peptide (IQR)	1029 (215–3378)	1184 (319–3270)	1550 (494–3430)	0.30
C-reactive protein mg/L (IQR)	17 (7–83)	15.5 (7–42)	20 (8–72)	0.05
Total cholesterol value mmol/L (IQR)	4 (3–5)	4 (3.4–4.9)	4 (3.1–4.7)	0.001
Triglyceride value mmol/L (IQR)	1.9 (1.1–2.3)	1.5 (1.1–2.2)	1.4 (1.0–2.0)	0.16
Low-density lipoprotein value mmol/L (IQR)	2.4 (1.7–3.2)	2.2 (1.6–3.0)	1.9 (1.4–2.8)	0.02
Troponin value ng/L (IQR)	40 (15–132)	31 (13–84)	31 (11–76)	0.07
Troponin peak ng/L (IQR)	158 (35–1917)	100 (27–758)	59 (18–387)	0.007
Creatine kinase-MB U/L peak (IQR)	5 (5–48)	5 (5–17)	5 (5–15)	0.15
LV Ejection fraction (EF) (IQR)	47 (37–54)	46 (37–55)	48 (39–54)	0.71

Continuous variables given as median and IQR: interquartile range; LV: left ventricular.

**Table 7:** Diagnosis of obese patients and procedures performed during hospital stay among obese patients.

Final Diagnosis	Grade I	Grade II	Grade III	*p* Value
Unstable angina	144 (9.3%)	52 (9.3%)	25 (6.5%)	0.20
NSTEMI	392 (25.4%)	130 (23.1%)	68 (17.6%)	0.005
STEMI	729 (47.2%)	209 (37.2%)	99 (25.6%)	0.001
Anterior MI	139 (9.0%)	33 (5.9%)	8 (2.1%)	0.001
Lateral MI	38 (2.5%)	10 (1.8%)	3 (0.8%)	0.10
Inferior MI	227 (14.7%)	56 (10.0%)	45 (11.4%)	0.009
Posterior MI	27 (1.7%)	5 (0.9%)	2 (0.5%)	0.09
Atrial fibrillation	95 (6.1%)	42 (7.5%)	44 (11.6%)	0.001
Stent restenosis	12 (0.8%)	2 (0.4%)	2 (0.5%)	0.53
Stent thrombosis	2 (0.1%)	1 (0.2%)	2 (0.5%)	0.31
Cardiomyopathy	134 (8.7%)	55 (9.8%)	42 (10.9%)	0.37
Shock	12 (0.8%)	8 (1.4%)	6 (1.6%)	0.25
Cardiac arrest	14 (0.9%)	9 (1.6%)	6 (1.6%)	0.31
Congestive heart failure	247 (16%)	121 (12.5%)	107 (27.6%)	0.001
Aortic regurgitation	1 (0.1%)	0 (0%)	1 (0.3%)	0.36
Aortic stenosis	28 (1.8%)	13 (2.3%)	14 (3.6%)	0.10
Mitral regurgitation	9 (0.6%)	10 (1.8%)	3 (0.9%)	0.03
Mitral stenosis	12 (0.8%)	6 (1.1%)	2 (0.5%)	0.64
Coronary angiographic findings				
Single vessel disease	251 (16.2%)	74 (13.2%)	30 (7.8%)	0.001
Two vessel disease	193 (12.5%)	42 (7.5%)	12 (3.1%)	0.001
Three vessel disease	227 (14.7%)	62 (11.0%)	37 (9.6%)	0.007
PCI at admission	286 (18.5%)	76 (13.5%)	22 (5.7%)	0.001
PCI before discharge	317 (20.5%)	81 (14.4%)	44 (11.4%)	0.001

PCI: percutaneous coronary intervention; MI: myocardial infarction; NSTEMI: non-ST-segment elevation myocardial infarction; STEMI: ST-segment elevation myocardial infarction.

**Table 8:** Complications during hospital stay among obese patients.

Complications	Grade I	Grade II	Grade III	*p* Value
Atrial fibrillation	46 (3.0%)	20 (3.6%)	15 (3.9%)	0.60
Ventricular tachycardia	27 (1.7%)	12 (2.1%)	0 (0%)	0.02
Ventricular fibrillation	16 (1.0%)	5 (0.9%)	2 (0.5%)	0.63
Cardiac arrest	21 (1.4%)	13 (2.3%)	7 (1.8%)	0.30
Left bundle branch block	9 (0.6%)	3 (0.5%)	1 (0.3%)	0.73
Right bundle branch block	11 (0.7%)	2 (0.4%)	0 (0%)	0.18
Congestive heart failure	35 (2.3%)	13 (2.3%)	6 (1.8%)	0.66
Intubation	28 (1.8%)	13 (2.3%)	9 (2.3%)	0.68
Cerebrovascular accident	1 (0.1%)	1 (0.2%)	1 (0.3%)	0.56
Shock	15 (1.0%)	6 (1.1%)	4 (1.0%)	0.98
Hospital stay in days (IQR)	3 (2–5)	3 (2–5)	4 (2–9)	0.53
Mortality	143 (9.3%)	63 (11.2%)	66 (17.1%)	0.001

**Table 9:** Multivariate regression analysis for predictors of mortality among obese patients unadjusted and adjusted for age, sex, and comorbidities.

Variable	Crude Odds Ratio	95% Confidence Interval	Adjusted Odds Ratio	95% Confidence Interval	*p* Value
Age in years	1.08	1.07–1.09	1.06	1.05–1.08	0.001
Gender (male vs. female)	0.40	0.36–0.44	0.88	0.67–1.15	0.09
Diabetes mellitus	3.34	2.94–3.80	1.75	1.23–2.50	0.001
Hypertension	3.36	2.94–3.83	1.27	0.86–1.87	0.23
* BMI >= 30	1.0	0.99–1.0	1.0	0.99–1.004	0.92
Variable					
Age in years	1.08	1.07–1.09	1.07	1.06–1.08	0.001
Gender (male vs. female)	0.40	0.36–0.44	0.85	0.70–1.04	0.11
Diabetes mellitus	3.34	2.94–3.80	1.67	1.34–2.06	0.001
Hypertension	3.36	2.94–3.83	1.30	1.04–1.64	0.02
** BMI >= 30 vs. < 30	1.47	1.25–1.74	1.04	0.87–1.25	0.67

* Continuous variable; ** categorical variable; BMI: body mass index.

## Data Availability

All data are given in the manuscript, tables, and figures.
